# Face-Specific Pupil Contagion in Infants

**DOI:** 10.3389/fpsyg.2021.789618

**Published:** 2022-01-05

**Authors:** Yuki Tsuji, So Kanazawa, Masami K. Yamaguchi

**Affiliations:** ^1^Research and Development Initiative, Chuo University, Tokyo, Japan; ^2^Department of Psychology, Japan Women’s University, Tokyo, Japan; ^3^Department of Psychology, Chuo University, Tokyo, Japan

**Keywords:** infant, dynamic change, pupil contagion, face specific, gaze perception

## Abstract

Pupil contagion is the phenomenon in which an observer’s pupil-diameter changes in response to another person’s pupil. Even chimpanzees and infants in early development stages show pupil contagion. This study investigated whether dynamic changes in pupil diameter would induce changes in infants’ pupil diameter. We also investigated pupil contagion in the context of different faces. We measured the pupil-diameter of 50 five- to six-month-old infants in response to changes in the pupil diameter (dilating/constricting) of upright and inverted faces. The results showed that (1) in the upright presentation condition, dilating the pupil diameter induced a change in the infants’ pupil diameter while constricting the pupil diameter did not induce a change, and (2) pupil contagion occurred only in the upright face presentation, and not in the inverted face presentation. These results indicate the face-inversion effect in infants’ pupil contagion.

## Introduction

An increasing body of evidence indicates that pupillary contagion is a social function that extends beyond mere physiological responses (Harrison et al., 2006; Kret et al., 2014, 2015; Prochazkova and Kret, 2017; Prochazkova et al., 2018). Pupil contagion is the phenomenon in which an observer’s pupil-diameter changes in response to another person’s pupil.

Recent research has also demonstrated that pupil contagion is found in infants (Fawcett et al., 2016, 2017). Fawcett et al. (2016) investigated whether observing schematic depictions of human eyes induces pupil contagion in 6- to 9-month-old infants. In their study, the infants viewed schematic depictions of eyes with smaller and larger pupils and their (observer infants) own pupil diameters were recorded. For both age groups, infants’ pupil diameter was greater when they viewed large-center circles than when they viewed small-center circles. Their study reported that infants at 4 and 6 months of age, when viewing the eye region of male and female adults with small, medium, or large pupils, showed pupil dilation in response to others’ large pupils, but not small or medium pupils (Fawcett et al., 2017). These studies showed that the schematic depictions of eyes or eye regions induces pupil contagion in infants and that infants’ pupil diameter dilate when infants observe stimuli with large pupils, whereas infants’ pupil diameter does not decrease when infants observe stimuli with small pupils. That is, pupil contagion shows an asymmetry between dilating and constricting pupil observation in infants.

Following the procedure of these studies, we examined this asymmetry in pupil contagion in dynamic changing pupil diameters in 5- to 6-month-old infants. A more recent study investigated infants’ pupillary mimicry responses in a wide age range of infants (Aktar et al., 2020). They used dynamically dilating and constricting stimuli, and measured pupil mimicry in infants and their parents. Their result indicated that the infants’ pupil response mimicked that of their parents. However, infants’ responses were slower than their parents’. They also showed that the pupil mimicry response in infants and adults was independent of race. However, as compared to our research, they only studied one eye region.

Aktar et al. (2020) also used dynamically changing pupil diameter, as perceiving or responding to pupil motion, as an important factor. One previous gaze study demonstrated that the motion of pupils induced infants’ attention (Farroni et al., 2000). Motion information generally enhances face and gaze recognition in young infants. Among the many objects infants perceive, faces are unique in that infants encounter faces that are nearly exclusive in motion. It seems likely, therefore, that facial motion seen in everyday life might promote infants’ ability to recognize faces. We believed that motion information would also be effective for infants’ pupil contagion.

In the present study, we also examined whether pupil contagion would occur in the context of the entire face rather than the eye region only. An adult fMRI study has shown the holistic processing of pupil contagion (Harrison et al., 2006). The authors investigated pupillary contagion and brain activity for the face stimuli with different emotional expressions (happiness, sadness, anger, and neutrality) and different pupil diameters. They found that mimicry of pupil diameter occurred in the context of sadness in the facial expression, as well as significant correlations between the participants’ individual sensitivity to pupillary contagion and activity in many of the regions [including the left frontal operculum, amygdala, superior temporal sulcus, and pupillary control nuclei (Edinger–Westphal)] in response to observed pupil diameter. In their experiment, faces with different expressions were used. Thus, their results might have another possible explanation for variations due to facial differences (Carsten et al., 2019). However, the most important point of their study is the finding regarding pupil contagion in the face context.

To investigate whether pupil contagion depends on the face context or not, we conducted an experiment using upright and inverted face to examine the face-inversion effect for pupil contagion. The face-inversion effect refers to the fact that faces are perceived and recognized more readily when presented upright than inverted. The Thatcher illusion is a famous phenomenon in which detecting local feature changes in an inverted face is difficult, whereas identical changes would be obvious in an upright face (Thompson, 1980). Studies have also found that faces are much more difficult to recognize upside-down compared with other kinds of objects (Yin, 1969). Many studies have been conducted on the face-inversion effect in infants. Simion et al. (2002) showed that newborn infants prefer a “top-heavy” configuration, that is, a geometric pattern that has more elements in the upper part than in the lower part of the configuration, and this preference disappeared upon inversion. Many electrophysiological and neuroimaging studies have investigated the neurofunctional mechanisms underlying the face-inversion effect. N170 is an early face-sensitive event-related potential component (Bötzel et al., 1995; Bentin et al., 1996). In previous studies, N170 was found to display longer latency and greater amplitude during the observation of inverted faces than when viewing upright faces (Watanabe et al., 2003; Honda et al., 2007). Functional magnetic resonance imaging (fMRI) studies using adaptation to facial identity reported a lower recovery from identity adaptation for inverted faces than for upright faces in the fusiform face areas (Yovel and Kanwisher, 2005; Mazard et al., 2006). On functional near-infrared spectroscopy (fNIRS) study, Otsuka et al. (2007) measured the hemodynamic responses of 5- to 8-month-old infants to images of upright and inverted faces in the temporal regions using fNIRS, and showed that the concentration of oxy-Hb and total-Hb in the right temporal region significantly increased for upright faces compared to inverted faces. These studies indicated the difficulty in processing inverted faces compared to upright faces. In the present study, we aimed to examine whether pupil contagion changes depending on the face orientation. Therefore, we experimented with investigating the face-inversion effect, as previously described in the studies mentioned above.

We aimed to determine whether dynamic changes in pupil diameter would induce changes in infants’ pupil diameter. We also investigated whether pupil contagion in infants depends on the face orientation. We measured infants’ pupil-diameter response to pupil change directions (dilating/constricting) in upright and inverted faces.

## Materials and Methods

### Participants

Fifty 5- and 6-month-olds infants (21 females, aged 147 – 194 days, mean age of 174 days) were recruited to the study, consisting of factors of face orientation (between-subject factor: upright vs. inverted) and pupil change direction (within-subject factor: dilating vs. constricting). The subjects were randomly assigned to the two face orientation conditions. An additional 27 infants participated but were not included in the final analyses owing to fussiness (*n* = 3 five-month-old and *n* = 2 six-month-old infants), machine trouble (i.e., inability to calibrate gaze; *n* = 7 five-month-old and *n* = 5 six-month-old infants), or insufficient data (*n* = 5 five-month-old and *n* = 5 six-month-old infants; at least six trials with 50% or more sampled pupil-diameter data per pupil change direction were required for inclusion). The infants were recruited through newspaper advertisements. All the infants were full-term at birth and healthy at the time of the experiment. Written informed consent was obtained from the parents of the participants. The study protocol was approved by the Ethical Committee of Chuo University. This study was conducted according to the principles and guidelines of the Declaration of Helsinki. Parents gave prior written informed consent for their children’s participation and for the publication of the results in an online open-access publication.

### Stimuli

To create a symmetrical eye region, we extracted one side of a face containing over 80% of the visible iris, which was vertically flipped and merged into a whole face. We cropped each face into an oval shape (15.7° × 18.5°). The distance between the left and right pupils in each face was 5.8 cm. To reduce the contrast at the boundaries between the face and background, we gradually changed the color from the face to the background (R, G, B = 171). We made the mean luminance [212 cd/m^2^ ± 0.58 (SD)] of the entire face uniform, except the irises and pupils. Average of the overall luminance of the stimuli was 213 cd/m^2^ ± 0.061 (SD). The eyes were then filled with new irises, whose color was gray (15 cd/m^2^) and an artificial pupil was added using GIMP ver2.10.4.

The artificial pupil diameter (5.0 mm) in each face was presented for 0.5 s, after which, in the constricting condition, the artificial pupil diameter was constricted by 60% (from 5.0 to 3.0 mm) for 2.5 s, while in the dilating condition, the artificial pupil diameter was dilated by 140% (from 5.0 to 7.0 mm) for 2.5 s (Kret et al., 2015; Prochazkova et al., 2018).

### Apparatus

The experimental stimuli were presented on a 23-inch LCD monitor (EIZO FlexScan EV2451, 1,920 × 1,080 pixel resolution, refresh rate of 60 Hz) using PsychoPy 3.0. Infants sat on their parents’ lap approximately 40 cm from the screen and eye tracker (Tobii pro spectrum; Tobii Technology, Inc., Danderyd, Sweden), which was used to record the infants’ eye movements. A camera (Logicool C920R) was set below the display to monitor and record the infants’ behavior while looking at the stimuli. An experimenter could observe the infants’ behavior through a monitor connected to the camera. A Tobii pro spectrum with a freedom of head movement within an area of 34 × 26 × 65 cm as used. Gaze was recorded at 150 Hz. Before beginning the experiment, a five-point calibration was conducted, with all points needing to be successfully calibrated.

### Procedure

**FIGURE 1:**
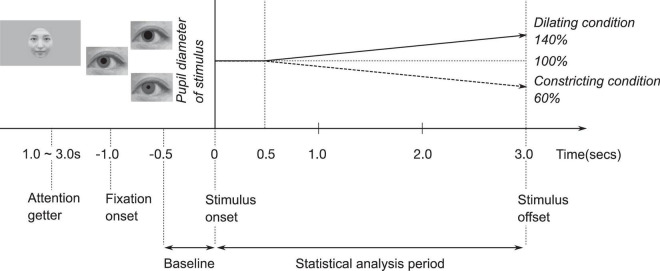
Experimental procedure.

The experiment was designed to measure two conditions: pupil change direction (dilating or constricting), as within-subject factors, and face orientation (upright or inverted) as the between-subjects factor. Six different female faces with identical pupil-diameter were presented in each condition. To call infants’ attention to the monitor, each trial was preceded by a short (1.0–3.0 s) animated video. The experimenter initiated each trial as soon as the infant began paying attention to the animated video. A fixation point in the form of a small black cross (2.38° × 2.38°) was presented for 1.0 s at the beginning of each trial. All stimuli, short animated videos and fixation point were presented on a gray background. In upright condition, each face (six female faces) was presented in the pupil-diameter dilating and constricting conditions for 3.0 s (Figure 1). Each condition was presented twice. The sequence of the presentation was pseudo-randomized. No more than two consecutive trials were ran for the same type of face and pupil change direction (dilating or constricting). The entire experimental session took approximately 2.5 min. In inverted condition, all faces were presented in upside-down.

### Data Analysis

Data files exported from the Tobii eye tracker were analyzed using MATLAB R2019a (The Mathworks, Natick, MA, United States). We excluded individual trials that were missing more than 50% of data in the combined baseline and trial (4 s) owing to inattention or technical problems. We analyzed the infants’ pupil diameter in a time series from −0.5 s to 3.0 s for each trial (Figure 1). The baseline was the average pupil diameter from −0.5 to 0 s. The baseline was subtracted from the pupil diameter for the statistical analysis period of 0 to 3.0 s. Gaps in the data for more than 15 samples were considered missing data, but smaller gaps were interpolated linearly. The data included gaps under 16 samples which were smoothed using a moving average over five samples.

In this study, we defined two areas of interest (AOIs). These were circles of 23.5 mm radius, centered on the left and the right pupil of the stimuli, which were used to assess infants’ attention on the eye region. Infants’ gazes often did not shift from the fixation cross during the trial. We calculated the percentage of their gaze on the AOIs and on the screen overall and compared the two. We calculated also looking time (ms) of their gaze on the AOIs. To examine the differences in percentages of the gaze on AOIs between conditions, we performed a two-way repeated measures analysis of variance (ANOVA), using pupil diameter change (dilating or constricting) as the within-subjects factor, and face orientation (upright or inverted) as the between-subjects factor.

We applied GLMM to analyze the data (Agresti, 2007; Bolker et al., 2009; Moscatelli et al., 2012). GLMM is an extension of the ordinary general linear model, which allows the analysis of clustered categorical data. We used the function glmer in the R (version 4.0.2; R Core Team, 2020) package lme4 (version 1.1.26) (Bates et al., 2015) for fitting GLMM. We initially included the following fixed effects in the model: time, looking time, pupil change direction (dilating or constricting), face orientation (upright or inverted), and all their interactions. Common across all models was that pupil-diameter change from baseline in individual trials was the dependent variable and that there were random intercepts for participants and trials. To obtain the most parsimonious model with the best fit, non-significant effects were removed one at a time, starting with the higher-order interactions. *Via* likelihood ratio tests, we verified whether the removal of a non-significant factor improved the fit of the model or not, in accordance with the most standard model-selection procedure. After specifying the fixed effects, we performed statistical tests of the variances of the random effects. To examine the differences in pupil diameter response between conditions, we performed a two-way repeated measures ANOVA, using pupil diameter change (dilating or constricting) as the within-subjects factor, and face orientation (upright or inverted) as the between-subjects factor.

## Results

We analyzed infants’ gaze and pupil-diameter responses to pupil change directions in upright faces and inverted faces.

### Areas of Interest

**FIGURE 2:**
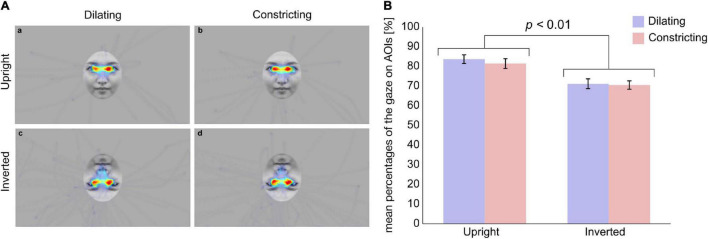
Average heat maps of infants’ gaze to stimuli and mean percentages of gaze on AOIs. **(A)** Average heat maps of infants’ gaze to stimuli in (a) the Dilating/Upright, (b) the Constricting/Upright, (c) the Dilating/Inverted, and (d) the Constricting/Inverted condition, respectively. White circles indicate AOIs. **(B)** Blue and red bars represent the dilating and constricting conditions, respectively. Error bars indicate standard errors. Mean percentage of the gaze on AOIs was higher in upright condition than in inverted condition (*p* < 0.01).

During the presentation of stimuli, 77% of the total recorded gaze was spent viewing AOIs. The mean percentages of the gaze on AOIs were 84% in the dilating pupil size on an upright face condition, 82% in the constricting pupil size on an upright face, 71% on the dilating pupil size on an inverted face, and 71% in the constricting pupil size on an inverted face condition (Figure 2). For mean percentages of the gaze on AOIs, a two-way ANOVA revealed a main effect of face orientation was significant (*F*_(1,48)_ = 18.1, *p* < 0.05, η^2^ = 0.27, Figure 2). There was no other significant main effect (pupil change direction: *F*_(1,48)_ = 0.768, *p* > 0.1, η^2^ = 0.016) or interaction (*F*_(1,48)_ = 0.224, *p* > 0.1, η^2^ = 0.0046). These results indicated that infant observed eye region on upright face more than that on inverted face.

### Pupil Diameter

To analyze infants’ (*n* = 50) pupil diameter in reaction to viewing the stimuli, we applied a GLMM. Initially, the GLMM was applied with time (ms), infants’ looking time (ms), pupil change direction (dilating or constricting), and face orientation (upright or inverted), and all their interactions as fixed effects, and with trial and individual differences as random effects. The likelihood ratio tests confirmed that the model with pupil change direction, face orientation and all their interactions as fixed effects provided a better fit to the data than the model with time, infants’ looking time, pupil change direction, face orientation and all their interactions as fixed effects (*p* < 0.01, see Supplementary Material). Thus, time and the interactions of time with any other effects were not included in the subsequent analyses.

The GLMM revealed two significant effects (pupil change direction: *b* = −0.070, *SE* = 0.024, *t* = −2.9, *p* = 0.0036, 95% CI = [−0.12, −0.023]; face orientation: *b* = −0.079, *SE* = 0.028, *t* = −2.9, *p* = 0.0043, 95% CI = [−0.13, −0.025]) and an interaction between pupil change direction and face orientation (*b* = 0.067, *SE* = 0.026, *t* = 2.6, *p* = 0.010, 95% CI = [0.016, 0.12]).

**FIGURE 3:**
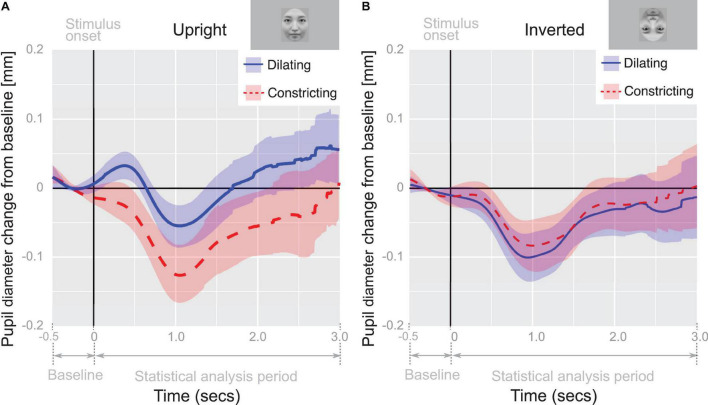
Pupil-diameter change from baseline. Recorded diameter of pupils over time while viewing pupil-diameter dilating or constricting (0–3 s) adjusted for baseline pupil diameter during fixation cross viewing (–0.5–0 s); The time course of the average change of pupil-diameter in panel **(A)** upright and **(B)** inverted face condition. Shaded regions indicate 95% confidence intervals.

In order to examine differences in pupil-diameter response between conditions, the mean pupil-diameter responses were further analyzed by performing a two-way repeated measures ANOVA, using pupil change direction (dilating or constricting) as the within-subjects factor and face orientation (upright or inverted) as the between-subjects factor. We identified a significant main effect of face orientation (*F*_(1,48)_ = 4.93, *p* < 0.05, η^2^ = 0.0932), pupil change direction (*F*_(1,48)_ = 19.0, *p* < 0.01, η^2^ = 0.284), and a significant interaction between factors (*F*_(1,48)_ = 20.0, *p* < 0.01, η^2^ = 0.294). *Post hoc* analysis (simple main effect tests) of the interaction revealed that the mean pupil diameter in the dilating condition was greater than that in the constricting condition when the face orientation was upright (*F*_(1,48)_ = 39.0, *p* < 0.01, η^2^ = 0.448, Figure 3). The mean pupil diameter in the dilating condition was also greater when the face orientation was upright compared to when it was inverted (*F*_(1,96)_ = 13.0, *p* < 0.01, η^2^ = 0.119). These outcomes provide further evidence that pupillary contagion occurs only in response to dilating pupils in an upright face.

## Discussion

In this study, we investigated whether dynamic changes in pupil diameter would induce changes in infants’ pupil diameter. We also investigated pupil contagion in the face orientation. To investigate these points, we measured the pupil-diameter response of 5- and 6-month old infants to pupil change direction (dilating/constricting) in upright and inverted faces. We hypothesized that dynamic changes in pupil diameter would induce the infants’ pupil contagion, whereas dynamic changes in pupil diameter in the inverted face condition would not. The results showed that the dynamic changes in pupil diameter induced infants’ pupil contagion and that pupil contagion occurred only in the upright face orientation, and not in the inverted face one. Additionally, infants’ pupil-diameter response differed between the pupil-diameter dilating and constricting conditions in the upright face orientation. This asymmetry in pupil contagion in our study was consistent with previous studies (Fawcett et al., 2016, 2017).

There is a possibility that the pupil contagion we observed might be attributed to luminance. The luminance of our experimental stimuli differed between the pupil-diameter dilating and constricting conditions. Infants’ pupil-diameter response (dilating/constricting) in our study might be due to the difference in luminance between the pupil-diameter changes (dilating/constricting). However, the luminance change in the pupil diameter was the same between the upright and inverted face conditions. In spite of the same luminance of the two conditions, infants’ pupil-diameter responses differed between the upright and inverted faces. Therefore, infants’ pupil-diameter response did not depend on the changing luminance of the pupil-diameter conditions in our study.

However, there is another way to interpret these pupil diameter responses. We interpreted the observed pupil-diameter response as a pupillary light response rather than pupil contagion. According to this point, we raise two possibilities. First, the pupillary light response is enhanced by focused attention (Binda et al., 2013; Mathôt et al., 2013; De Winter et al., 2021). Derksen et al. (2018) conducted several experiments with luminance-controlled and luminance-not-controlled stimuli of static and dynamic pupils of various sizes, and indicated that the pupil contagion phenomenon occurs due to luminance and participants’ attention shift toward the eye region. This result suggests subtle differences in attention-dependent pupil responses to luminance changes in the face area (Mathôt and Naber, 2018). This focused attention is associated with social aspects, such as facial preference or attractiveness (Birmingham and Kingstone, 2009), in-group members (Kret et al., 2015), as well as empathy (Harrison et al., 2006, 2009). Second, the pupillary light response is weakened by the inversion effect in various images (Naber and Nakayama, 2013). For example, Naber and Nakayama (2013) demonstrated that pupillary light responses for natural and artificial scenes were weakened by upside-down. However, previous studies have shown that face-inversion induces electroencephalography and hemodynamic responses in the face area. This indicates that face-inversion can induce a social response. Such a social aspect of face-inversion might affect the pupillary light response.

We found the face-inversion effect in infants’ pupil contagion. This result suggests the possibility that presenting faces in a different context would affect pupil contagion. In general, the face had been considered a social stimulus. This is not in line with the result of Carsten et al. (2019), who demonstrated that the degree of pupillary contagion was independent of emotional expression, as mentioned in the introduction. The contradiction is that Carsten et al. (2019) showed that pupil contagion is independent of emotion. Conversely, we showed that pupil contagion is dependent on the face that is a social stimulus. However, infants may interpret faces as shapes, and not as social stimuli. Studies on newborns have consistently showed that geometric face patterns grab the infants’ attention. For example, the famous “top-heavy” configuration has been shown to attract the attention of newborns, meaning that newborns prefer certain facial geometric patterns (Simion et al., 2002). Furthermore, Batki et al. (2000) showed that newborns spend more time looking at faces with open eyes than at faces with closed eyes. This showed that eyes, when combined with the geometric face pattern, serves as an important cue to grab the infants’ attention. Therefore, we believe that our findings regarding pupil contagion suggest that pupil contagion occurs according to the context of the facial geometric pattern.

Another possibility was that simple physiological preference induces infants’ pupil-diameter dilating response. For example, a mother’s face induces infants’ pupil diameter to dilate (Fitzgerald, 1968). In general, expansion, but not contraction, stimulus also induces infants’ preference (Shirai et al., 2004). This expansion preference might induce infants’ pupil-diameter dilating response to pupil-diameter dilating. That is, the physical expansion of the black circle of the pupil might induce infants’ pupil-diameter dilating response. However, the physical expansion of the black circle (pupil) was the same between the upright and inverted face conditions. In spite of the same physical differences in the dilating pupil, the infants’ pupil-diameter response differed between the upright and inverted face conditions. Therefore, infants’ pupil-diameter response did not depend on the physical dilating.

## Conclusion

Our study investigated 5- and 6-month-old infants’ pupil contagion. We found that in the upright face presentation, presenting a dilating pupil-diameter induced a change in the infants’ pupil diameter. However, constricting the pupil-diameter did not induce a response. Pupil contagion did not occur in the inverted face presentation. These results indicated the face-inversion effect in infants’ pupil contagion. They suggest that pupil contagion occurs according to the face orientation.

## Data Availability Statement

The raw data supporting the conclusions of this article will be made available by the authors, without undue reservation.

## Ethics Statement

The studies involving human participants were reviewed and approved by the Ethical Committee of Chuo University. Written informed consent was obtained from the participants’ legal guardian/next of kin for the publication of any potentially identifiable images or data included in this article.

## Author Contributions

YT, SK, and MY designed the experiments and wrote the manuscript. YT performed the experiments and analyzed the data. All authors contributed to the article and approved the submitted version.

## Conflict of Interest

The authors declare that the research was conducted in the absence of any commercial or financial relationships that could be construed as a potential conflict of interest.

## Publisher’s Note

All claims expressed in this article are solely those of the authors and do not necessarily represent those of their affiliated organizations, or those of the publisher, the editors and the reviewers. Any product that may be evaluated in this article, or claim that may be made by its manufacturer, is not guaranteed or endorsed by the publisher.
