# Supplementary material for: Face-Specific Pupil Contagion in Infants
Source: Front Psychol. 2022 Jan 5;12:789618. doi: 10.3389/fpsyg.2021.789618 (PMC8767059; doi:10.3389/fpsyg.2021.789618)
Supplement: Supplementary file 1 [file Table_1.DOCX]

Supplementary Material

# Results of GLMM model-selection

We initially included the following fixed effects in the Model0: time, looking time, pupil change direction (dilating or constricting), face orientation (upright or inverted), and all their interactions. The GLMM results indicated that no factor had a significant effect on pupil-diameter. To obtain the most parsimonious model with the best fit, non-significant effects were removed one at a time, starting with the higher-order interactions to single terms. Via likelihood ratio tests, we verified whether the removal of a non-significant factor improved the fit of the model or not, in accordance with the most standard model-selection procedure.

In Model0, the highest-order interaction which was not significant was 4-way interaction of time, looking time, pupil change direction, and face orientation (*b* = -0.20, *SE* = 0.22, *t* = -0.93, *p* = 0.35, 95% CI = [-0.63 0.22]). We tested simpler model by dropping this interaction (Model1) and applying likelihood ratio tests. The likelihood ratio test revealed that removal of this 4-way interaction factor improved fit of the model (χ2 = 3.3×10^3^, *df* = 33, *p* < 0.01).

In Model1, the highest-order interaction which was not significant was 3-way interaction of time, pupil change direction, and face orientation (*b* = 0.0021, *SE* = 0.23, *t* = 0.090, *p* = 0.93, 95% CI = [-0.043 0.048]). We tested simpler model by dropping this interaction (Model2) and applying likelihood ratio tests. The likelihood ratio test revealed that removal of this 3-way interaction factor improved fit of the model (χ2 = 3.5×10^3^, *df* = 31, *p* < 0.01).

In Model2, the highest-order interaction which was not significant was 3-way interaction of time, looking time, and pupil change direction (*b* = 0.00023, *SE* = 0.014, *t* = 0.016, *p* = 0.99, 95% CI = [-0.028 0.028]). We tested simpler model by dropping this interaction (Model3) and applying likelihood ratio tests. The likelihood ratio test revealed that removal of this 3-way interaction factor improved fit of the model (χ2 = 2.6×10^3^, *df* = 29, *p* < 0.01).

In Model3, the highest-order interaction which was not significant was 3-way interaction of looking time, pupil change direction, and face orientation (*b* = -0.46, *SE* = 0.86, *t* = -0.53, *p* = 0.6, 95% CI = [-2.1 1.2]). We tested simpler model by dropping this interaction (Model4) and applying likelihood ratio tests. The likelihood ratio test revealed that removal of this 3-way interaction factor improved fit of the model (χ2 = 8.6×10^3^, *df* = 27, *p* < 0.01).

In Model4, the highest-order interaction which was not significant was 2-way interaction between time and pupil change direction (*b* = -0.010, *SE* = 0.0079, *t* = -1.3, *p* = 0.20, 95% CI = [-0.026 0.0053]). We tested simpler model by dropping this interaction (Model5) and applying likelihood ratio tests. The likelihood ratio test revealed that removal of this 2-way interaction factor improved fit of the model (χ2 = 2.7×10^3^, *df* = 25, *p* < 0.01).

In Model5, the highest-order interaction which was not significant was 3-way interaction of time, looking time, and face orientation (*b* = -0.049, *SE* = 0.028, *t* = -1.7, *p* = 0.083, 95% CI = [-0.10 0.0065]). We tested simpler model by dropping this interaction (Model6) and applying likelihood ratio tests. The likelihood ratio test revealed that removal of this 3-way interaction factor improved fit of the model (χ2 = 7.0×10^3^, *df* = 23, *p* < 0.01).

In Model6, the highest-order interaction which was not significant was 2-way interaction between looking time and face orientation (*b* = 0.00070, *SE* = 0.037, *t* = 0.019, *p* = 0.98, 95% CI = [-0.072 0.073]). We tested simpler model by dropping this interaction (Model7) and applying likelihood ratio tests. The likelihood ratio test revealed that removal of this 2-way interaction factor improved fit of the model (χ2 = 8.1×10^3^, *df* = 21, *p* < 0.01).

In Model7, the highest-order interaction which was not significant was 2-way interaction between time and face orientation (*b* = -0.020, *SE* = 0.017, *t* = -1.2, *p* = 0.24, 95% CI = [-0.054 0.014]). We tested simpler model by dropping this interaction (Model8) and applying likelihood ratio tests. The likelihood ratio test revealed that removal of this 2-way interaction factor improved fit of the model (χ2 = 1.1×10^4^, *df* = 19, *p* < 0.01).

In Model8, the highest-order interaction which was not significant was 2-way interaction between time and looking time (*b* = -0.0096, *SE* = 0.010, *t* = -0.94, *p* = 0.35, 95% CI = [-0.030 0.010]). We tested simpler model by dropping this interaction (Model9) and applying likelihood ratio tests. The likelihood ratio test revealed that removal of this 2-way interaction factor improved fit of the model (χ2 = 5.7×10^3^, *df* = 17, *p* < 0.01).

In Model9, the highest-order interaction which was not significant was 2-way interaction between looking time and pupil change direction (*b* = 0.037, *SE* = 0.032, *t* = 1.2, *p* = 0.25, 95% CI = [-0.025 0.099]). We tested simpler model by dropping this interaction (Model10) and applying likelihood ratio tests. The likelihood ratio test revealed that removal of this 2-way interaction factor improved fit of the model (χ2 = 1.0×10^4^, *df* = 15, *p* < 0.01).

In Model10, the looking time factor was not significant (*b* = 0.016, *SE* = 0.016, *t* = 1.0, *p* = 0.32, 95% CI = [-0.016 0.048]). We tested simpler model by dropping this factor (Model11) and applying likelihood ratio tests. The likelihood ratio test revealed that removal of this factor improve model (χ2 = 9.6×10^3^, *df* = 13, *p* < 0.01).

In Model11, the time factor was not significant (*b* = 0.011, *SE* = 0.0071, *t* = 1.6, *p* = 0.11, 95% CI = [-0.0026 0.025]). We tested simpler model by dropping this factor (Model12) and applying likelihood ratio tests. The likelihood ratio test revealed that removal of this factor improve model (χ2 = 1.0×10^4^, *df* = 11, *p* < 0.01).

Model12 included the following fixed effects: pupil change direction (dilating or constricting), face orientation (upright or inverted), and their interactions. The GLMM revealed two significant effects (pupil change direction: *b* = -0.070, *SE* =0.024, *t* =-2.9, *p* =0.0036﻿, 95% CI = [-0.12, -0.023]; face orientation: *b* =-0.079, *SE* = 0.028, *t* = -2.9, *p* = 0.0043, 95% CI = [-0.13 -0.025]) and an interaction between pupil change direction and face orientation (*b* = 0.067, *SE* = 0.026, *t* = 2.6, *p* = 0.010, 95% CI = [0.016 0.12]). Therefore, we decided that the most parsimonious model with the best fit was Model12.
